# Enhancing Corneal Sensitivity in Diabetic Patients Through an Innovative Ophthalmic Solution: In Vivo and Vitro Results

**DOI:** 10.3390/jcm14010245

**Published:** 2025-01-03

**Authors:** Fabio Scarinci, Giovanna De Simone, Chiara Ciancimino, Claudio Caggiano, Giulio Pocobelli, Alessandra Di Masi

**Affiliations:** 1 Ophthalmology, San Giovanni-Addolorata Hospital, Via Santo Stefano Rotondo, 6, 00184 Rome, Italy; 2 Department of Sciences, Section of Biomedical Sciences and Technologies, Roma Tre University, Viale Marconi 446, 00146 Rome, Italy; 3 Ophthalmology Unit, Department of Neurosciences, Mental Health and Sensory Organs (NESMOS), St. Andrea Hospital, “Sapienza” University of Rome, Via di Grottarossa 1035/1039, 00189 Rome, Italy; 4 One Day Medical Center, Via Ambrosini 114, 00147 Rome, Italy; 5 Department of Microbiology, Immunology, Infectious Diseases, and Transplants (MIMIT), University of Rome Tor Vergata, 00133 Rome, Italy

**Keywords:** wound healing, corneal sensitivity, diabetes, ocular surface, tears

## Abstract

**Background/Objectives**: Diabetes is a well-recognised factor inducing a plethora of corneal alterations ranging from dry eye to reduced corneal sensibility, epithelial defects, and reduced cicatrisation. This cohort study aimed to assess the efficacy of a novel ophthalmic solution combining cross-linked hyaluronic acid (CHA), chondroitin sulfate (CS), and inositol (INS) in managing diabetes-induced corneal alterations. Specifically, it evaluated the solution’s impact on the tear breakup time (TBUT), the ocular surface disease index (OSDI), and corneal sensitivity after three months of treatment. Additionally, the solution’s potential to promote wound healing was examined. **Methods**: Two different populations were retrieved from the database; the first one was composed of 20 diabetic subjects treated for three months with the ophthalmic CAH-CS (OPHTAGON srl, Rome, Italy), while the second group was composed of 20 diabetic subjects who did not want to use any eye lubricant or other treatment. The outcome measures were the TBUT, the OSDI score, and the corneal sensitivity measured using a Cochet–Bonnet aesthesiometer. To investigate the wound-healing properties, in vitro tests were conducted using two cell lines, comparing the results of scratch tests with and without the solution. **Results**: The results indicate that CHA-CS significantly improved the tear film stability, as evidenced by an increased TBUT and a reduction in dry eye symptoms reflected by lower OSDI scores. Moreover, the solution was associated with an enhanced corneal sensitivity in treated patients. In wound-healing assays, CHA-CS promoted cell motility, suggesting a supportive role in tissue repair compared to untreated cells. **Conclusions**: Collectively, the results suggest that CHA-CS could serve as an innovative tool for the treatment of diabetic patients with corneal alterations and delayed corneal sensitivity. Clinical trial registration number: Clinical Trial.gov NCT06573606.

## 1. Introduction

Diabetes is a chronic condition with a systemic impact resulting in a plethora of complications, including ocular ones, such as diabetic keratopathy and retinopathy, cataracts, glaucoma, and dry eye disease (DED) [1]. The cornea, a highly innervated ocular layer, is one of the eye structures most affected by diabetes [2]. The well-being of the ocular surface relies on the preserved structure of corneal nerves, which are responsible for various homeostatic functions, including blinking and the release of neuropeptides, neurotrophins, and growth factors that are directly involved in the corneal epithelial function and regeneration [3,4]. Therefore, the increasing incidence of diabetes mellitus has significant implications for ocular health, particularly in diabetic keratopathy.

Diabetic keratopathy represents a significant, yet often overlooked, complication stemming from diabetes mellitus. The prevalence of this condition is alarmingly high, affecting approximately 46–64% of diabetic patients, which can lead to serious ocular complications, including persistent epithelial defects and corneal infections that may necessitate surgical interventions, such as corneal transplantation [4,5]. The pathophysiology involves complex interactions between hyperglycaemia-induced nerve damage and structural changes in the cornea, exacerbated by a decreased corneal sensitivity, which heightens the risk of trauma and delayed wound healing [2,6]

Chronic hyperglycaemia plays a pivotal role in the degeneration of corneal nerves, since it induces polyol cycle disruption and inositol depletion, perpetuating a cycle of oxidative stress, nerve degeneration, and the accumulation of advanced glycation end-products (AGEs), which hinder the growth factor efficacy essential for epithelial repair and nerve function and regeneration [7]. Indeed, sustained hyperglycaemia leads to the conversion of excess glucose into sorbitol through the polyol pathway, facilitated by the enzyme aldose reductase. This process contributes to osmotic and oxidative stress and results in the depletion of inositol, a vital molecule for neuronal health and function. The reduced levels of inositol impair crucial signalling pathways, including the PI 3-kinase/Akt pathway, which is essential for the regeneration of nerves and the proper response of the cell to the insulin and insulin-like growth factor family [7], the members of which are important for corneal integrity [6]. In addition, studies indicate that the loss of corneal-nerve-produced trophic factors, such as the brain-derived neurotrophic factor (BDNF), due to impaired nerve function exacerbates neuronal dysfunction. In contrast, a diminished corneal sensitivity can directly lead to neurotrophic keratopathy [6,8]. Furthermore, alterations in the epidermal growth factor receptor (EGFR) signalling pathway due to hyperglycaemia contribute to delayed wound healing, complicating treatment approaches [9]. Consequently, patients frequently experience uncomfortable and debilitating symptoms like persistent epithelial defects, heightening the risk for serious complications, including corneal ulcerations and potential vision loss [5]. Hence, restoring sensitivity through innovative topical treatments that might address poor ocular drug retention [10] or specific etiopathogenetic factors represents a promising avenue to mitigate these effects and circumvent the necessity for more invasive interventions, such as corneal transplantation, which is both costly and fraught with risk [9].

Indeed, the same etiopathogenetic pathway has been proven to be responsible for diabetic peripheral neuropathy (DPN). DPN is defined as the presence of symptoms and/or signs of peripheral nerve dysfunction in individuals with diabetes, after excluding other potential causes [11]. DPN is characterised by pain, paraesthesia, and sensory loss, affecting around 50% of diabetics. It is associated with significant morbidity, mortality, and a reduced quality of life [12]. DPN begins in the toes and progressively moves upward. Once established in the lower limbs, it then affects the upper limbs, with sensory loss following the typical ‘glove and stocking’ distribution pattern [13]. Sensory loss occurs due to the disruption of the polyol pathway, which leads to sensory neurons becoming insulin resistant [14], and therefore, not being able to respond to insulin [15,16]; neuron apoptosis (a reduction in the number of neurons); and the apoptosis of glial cells (resulting in slower signal transduction and a diminished functionality). These effects have also been confirmed at the corneal level using corneal confocal microscopy. Diabetic subjects experience a decrease in both the number of neurons and the branching of corneal nerves [8].

The literature data have shown, both in vitro and in vivo, that, by restoring the polyol pathway, and therefore restoring the proper osmotic balance, it is possible to improve signal transduction. Indeed, researchers have been able to prove that there is a significant inositol reduction in the sciatic nerve of diabetic rats and a linked reduced nerve conduction velocity, and by restoring the polyol pathway, the nerve conduction velocity returned to an almost normal value in 14 days [17]. Additional in vitro data have shown that, by restoring insulin signalling, mitochondrial ATP production was more efficient (i.e., less oxidative stress) and the number of neurites was increased [16]. In addition, it has been proven that oral inositol administration is able to restore nerve conduction velocity [13,18].

Of note, by increasing inositol ingestion in human diabetic subjects, the nerve conduction velocity increased [18]. A diminished corneal sensitivity poses significant risks, particularly in the context of potential corneal transplantation. The intricate relationship between corneal innervation and wound healing underscores the necessity of restoring nerve function to avert the complications associated with surgical interventions. Research indicates that an impaired corneal nerve integrity contributes to delayed wound healing and an increased susceptibility to infections, jeopardizing graft success rates [4,19]. Moreover, the absence of appropriate sensory feedback exacerbates the risk of traumatic injuries, further complicating surgical outcomes.

Another important aspect that needs to be mentioned is that nerve defects play an important role in severe presentations of diabetic dry eye [20]. The prevalence of dry eye in diabetes is 15–33% in those over 65 years of age and is 50% more common in women than in men. Dry eye in diabetes can also develop following several ocular surgeries such as cataract surgery, photorefractive keratectomy, and LASIK, complicating the treatment of diabetes-induced corneal erosions and epithelial defects [4]. For this reason, the chosen therapeutic approach must target both DED-like symptoms and corneal sensitivity.

Based on the above, the present manuscript aimed to investigate the potential benefit of a formulation containing a combination of cross-linked hyaluronic acid (CHA) [21,22,23], chondroitin sulphate (CS) [24], and inositol (INS) to improve the corneal sensitivity in diabetic patients. Indeed, while INS was expected to improve the corneal sensitivity, CHA and CS were expected to reduce the DED-like symptoms induced by the nerve defect. To corroborate this hypothesis, the potential capability of this new solution in wound healing was also tested in vitro.

## 2. Materials and Methods

### 2.1. Study Design and Participants

This cohort observational study evaluated the clinical results obtained on diabetic patients diagnosed with dry eye disease with an age between 44 and 84 years old. The data were collected from diabetic patients who were counselled during routine clinical check-ups as part of standard clinical practice. The patients had well-controlled diabetes (i.e., an HbA1c < 7%) for at least 10 years and the fundoscopic exam showed no signs of proliferative diabetic retinopathy. All the patients provided written informed consent; the study adhered to the Declaration of Helsinki (1964). Subjects with an HBA1C ≥7% and with signs and symptoms of proliferative diabetic retinopathy were excluded. Clinical trial registration number: Clinical Trial.gov NCT06573606.

### 2.2. Treatment

Among the study cohort, two subgroups were analysed: one that agreed to use eye drops to treat dry eye disease (DED) symptoms and another subgroup that preferred not to use any lubricant eye drops or any other treatment. In the first subgroup, we were able to identify twenty diabetic patients (40 eyes; mean age, 65 ± 11; 65% females, 35% males) who were treated with the commercially available CHA-CS eye drops (OPHTAGON srl, Rome, Italy): two drops were administered three or four times a day for 3 months, a dosage treatment in line with what has been approved by the regulatory authorities for this product. Any adverse events or complications that appeared during the study were recorded to evaluate the safety of these treatments. In the second cohort subgroup, which was used as a control group, 40 eyes were enrolled (matching for gender and age).

### 2.3. Outcome Measures

A complete eye examination was carried out before the enrolment and during the follow-up to define corneal injuries in the enrolled patients. A corneal sensitivity analysis was performed using a Cochet–Bonnet aesthesiometer (CB-A) (C.O.I. Compagnia Ottica Italiana Srl, Milan, Italy) before (T0) and after (T3) 3 months of CHA-CS treatment. With this instrument, a mechanical stimulus is delivered using hair or nylon filaments of a variable diameter and length. The stimulus pressure applied is inversely proportional to the filament length, assuming a reproducible amount of bend in the filament [25]. The subjects report when they can feel the thread touching the ocular surface, and the length of thread at which this occurs is recorded. This length is converted into pressure using a calibration curve and the reciprocal of this value gives the corneal sensitivity. The test is performed on 4 different quadrants [26]. To evaluate the efficacy of CHA-CS for the treatment of dry eye symptoms, quantitative and qualitative data, including the tear film break-up time (TBUT) and the ocular surface disease index (OSDI), were assessed before (T0) and after (T3) 3 months of treatment. In particular, the TBUT test was performed by REmark^®^ (SERVIMED spa, Rome, Italy). During the test, fluorescein dye was instilled into the tear film. The patient was asked not to blink, and the time between the last blink and the first appearance of a dry spot on the cornea was measured under cobalt blue light. A TBUT of less than 10 s is generally considered abnormal, indicating tear film instability and potential dry eye disease [27]. The OSDI score, consisting of 12 questions that evaluate symptoms related to ocular discomfort, visual function, and environmental triggers, was assessed, with a threshold of ≥13 for inclusion indicating the presence of clinically relevant symptoms. The OSDI score was scored on a scale of 0 to 100, with higher scores representing a more severe disease and a negative change from baseline, demonstrating improvement [28].

### 2.4. Cell Lines and Culture Conditions

To assess CHA-CS’s ability to improve wound healing, we decided to use two different cell lines: the A549 (lung carcinoma epithelial cells; ATCC accession number CCL-185) cell line was obtained from the American Type Culture Collection (ATCC) (Manassas, VA, USA), whereas the MRC-5 (foetal lung fibroblast cells immortalized with SV-40; ATCC accession number CCL-171) cell line was purchased from Coriell Institute (Coriell Institute, Camden, NJ, USA).

The A549 and MRC-5 cells were cultured in Dulbecco’s modified Eagle medium (DMEM) (D6429, Corning, NY, USA) complemented with 10% foetal bovine serum (FBS) (35-079-CV, Corning), 100 μg/mL of penicillin and streptomycin (30-002-CI, Corning, NY, USA), and 2.0 × 10^−3^ M L-glutamine (25-005-CI, Corning, NY, USA) at 37 °C and 5% CO_2_.

The selection of the two cell lines for the wound-healing experiments was based on lineages present in the eye (i.e., epithelial cells and fibroblasts) capable of proliferating without the addition of growth factors, and for which well-established and extensive studies on proliferation and migration kinetics were available.

### 2.5. Wound-Healing Assay

To simulate cell wounding, Ibidi Culture-Inserts 2 Well (81176, Ibidi cell focus, Fitchburg, WI, USA) were used. These inserts are characterised by 2 silicon wells separated by a 500 µm gap, creating a cell-free region. After placing the insert in the centre of a 24-multiwell plate, 70 µL of a complete medium containing 4.5 × 10^5^ cells (either A549 or MRC-5) was added to each of the two silicon wells. After 24 h, the Culture-Insert 2 Well was gently removed using sterile tweezers. Subsequently, the cells were treated with 1.0 mL of a CHA-CS solution; cells treated with 1.0 mL of a complete medium complemented with 5% FBS were used as a control group. The imaging acquisition started immediately after wound application and continued for 30 h, or at least until complete cell confluence. The experiments were performed in duplicate for each cell line and treatment.

### 2.6. Image Acquisition

Image acquisition was performed under 10/0.22× magnification using a Leica DM IL LED microscope (Leica, Wetzlar, Germany) equipped with a Samsung camera. An illumination adjustment was carried out before the plate was inserted into the microscope stage, and the acquisition positions were set under the control of the camera immediately after the scratch was applied. The acquired images were subsequently processed using Adobe Photoshop (San Jose, CA, USA) to obtain area values to study the wound-closure dynamics.

### 2.7. Migration Quantification Methods

The quantification of cell migration in the wound-healing assay was performed by the area method [29]. The migration rate was indirectly evaluated as the percentage of wound area at a specific time point according to the following equation:

$$A_{t}=\frac{A_{0}-A_{t}}{A_{0}}\times 100$$

where A_t_ is the wound area at time t and A_0_ is its initial area.

### 2.8. Wound-Healing Velocity Calculation

To calculate the mean velocity of wound edges, the displacement of the area between sequential data points was divided by the length of the wound. Then, it was divided by 2 to determine the average speed of cells at each side of the wound [30].

### 2.9. Data Analysis

The population data, TBUT, OSDI, and corneal sensitivity results are shown as the mean ± standard deviation (SD). The wound-healing results are shown as the mean ± SD, derived minimally from 2 independent experiments. The statistical significance between means, assessed by a *t*-test or Wilcoxon test when appropriate (GraphPad InStat 3.1 Software Inc., San Diego, CA, USA), was considered significant when the *p*-values were ≤0.05.

## 3. Results

### 3.1. In Vivo Results

#### CHA-CS Treatment Improves Corneal Sensitivity, TBUT, and OSDI

**Figure 1:**
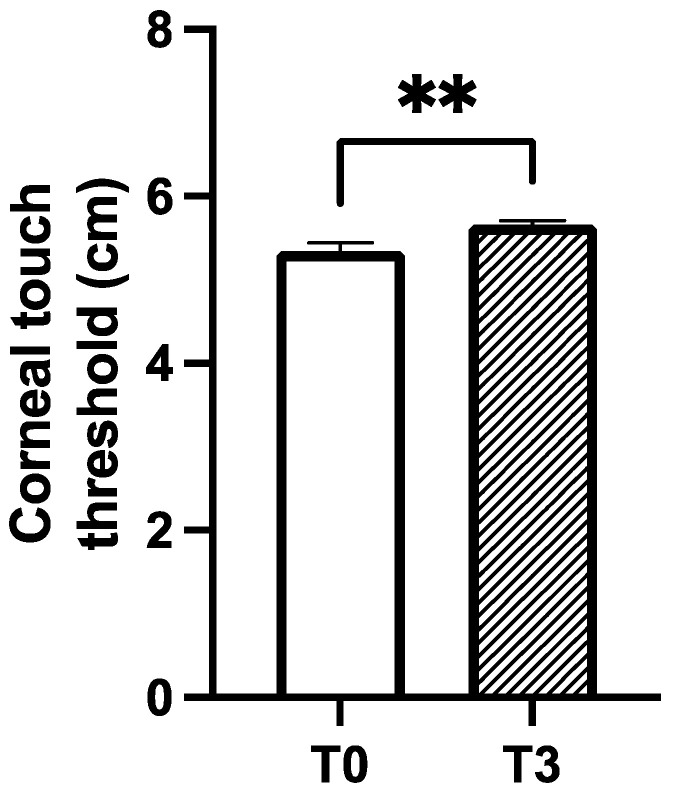
Corneal sensitivity was measured before (T0) and after (T3) 3 months of treatment with CHA-CS. Corneal touch threshold was measured in centimetres (cm); ** *p*-value < 0.01 vs. T0 (Wilcoxon matched-pairs signed-rank test).

In the first subgroup, which comprised 40 eyes from 20 diabetic patients with diabetes-induced dry eye disease (DED), the mean age of the patients was 65.2 ± 10.6 years (mean ± SD). At the end of the 3-month treatment period (T3), statistically significant improvements were observed in the corneal sensitivity, TBUT, and OSDI parameters compared to the initial measurements (T0). At T3, the diabetic patients showed a significant increment in the corneal sensitivity of about 5.5% (from 5.3 ± 0.6 to 5.7 ± 0.3), as displayed by the increased corneal touch threshold (*p* < 0.01; Figure 1).

**Figure 2:**
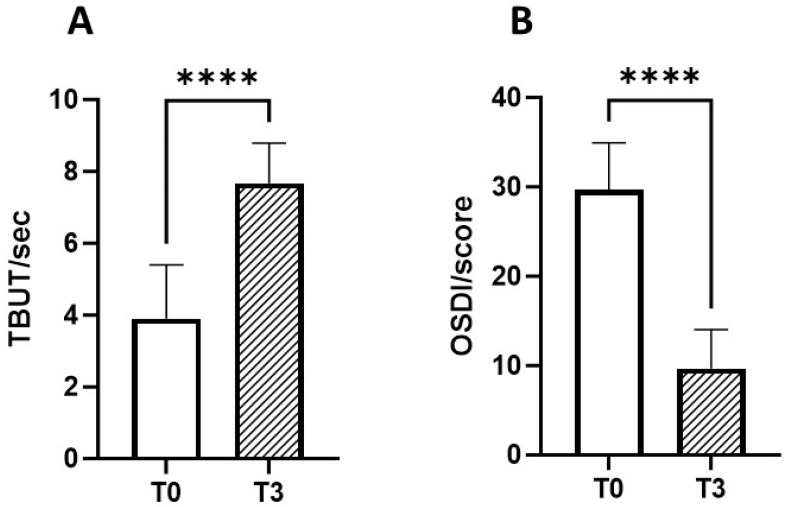
Subgroup one: TBUT and OSDI measured before (T0) and after (T3) 3 months of treatment with CHA-CS. (**A**) The stability of the tear film, measured as the time in seconds (sec) taken for the first dry spot to appear on the cornea after a complete blink at T0 and T3. (**B**) The OSDI score at T0 and T3. **** *p*-value < 0.0001 (Wilcoxon matched-pairs signed-rank test).

At T3, the TBUT score increased by roughly 49.4% (from 3.9 ± 1.5 to 7.7 ± 1.1) (*p* < 0.0001; Figure 2A). Similarly, at T3, the mean OSDI score decreased by about 67.3% (from 29.7 ± 5.3 to 9.7 ± 4.4) (*p* < 0.0001; Figure 2B).

At the same time, the second subgroup (40 eyes) who refused to use eye drops had a TBUT of 3.79 ± 2.5 and an OSDI of 31.2 ± 3.3 at baseline, while the following check-up did not show any significant changes.

### 3.2. In Vitro Results

#### CHA-CS Promotes Wound Healing

In both the A549 and MRC5 cells, wound closure occurred at a nearly constant rate from the first hour after scratching. In the presence of the CHA-CS solution, both the A549 and the MRC5 cells exhibited significantly increased wound closure by 1.08-fold with respect to the control. Similarly, the treatment of MRC5 cells with CHA-CS exhibited increased wound closure by 1.1-fold compared to the control (Table 1 and Figure S1).

## 4. Discussion

The present study showed the potential benefit of the daily use of a novel ophthalmic solution containing CHA, CS, and INS in ameliorating corneal alterations induced by diabetes, through an observational analysis and in vitro experiments.

Treatment with CHA-CS resulted in significant improvements in both objective and subjective measurements of the corneal health in diabetic patients. Indeed, the collected data clearly demonstrate an improvement in the tear film stability (TBUT) and a decrease in ocular surface discomfort (OSDI scores). The treatment also led to an enhanced corneal sensitivity, and it is interesting to speculate on a subsequent potential improvement in the neuroregulatory mechanisms, which are fundamental for maintaining corneal health. Also, our in-vitro findings support our hypothesis that the combined action of CHA, CS, and INS may contribute to the natural processes involved in corneal wound healing.

One of the most innovative aspects of the CHA-CS formulation lies in the presence of biotechnologically derived CS. This offers increased stability and biocompatibility and the avoidance of immunogenicity issues associated with animal-derived counterparts, thus ensuring an improved product safety and efficacy profile. The synergistic interaction between the CS and CHA molecules played a crucial role in alleviating the symptoms of DED in diabetic patients, as evidenced by the improvement in the TBUT values and OSDI scores observed following treatment with CHA-CS. CS, a natural glycosaminoglycan, is known for its strong hydration and protective properties. It is expressed in corneal and conjunctival epithelia and functions as a boundary lubricant: its deficiency causes the dry eye phenotype. Therefore, CS provides an additional layer of lubrication and acts as a protective barrier on the ocular surface, which can shield the corneal epithelium from mechanical stress and promote cell regeneration [31,32,33]. Additionally, CS seems to be a pivotal component of the extracellular matrix, characterising the limbal stem cell niche, further supporting the notion that the CHA-CS formulation supports epithelial wound healing [34]. On the other hand, CHA adds to the formulation’s viscoelasticity and stability, forming a gel-like layer that mimics the natural tear film’s viscosity. This property allows for prolonged retention on the ocular surface, providing extended hydration and facilitating tear film restoration. Based on this, in the CHA-CS formulation, CHA and CS enhance the viscoelasticity, stability, and mucoadhesivity of the solution [23,35], promoting tear film restoration, which is essential for maintaining corneal health [24,36,37]. These findings are consistent with those reported in previous literature [38].

Despite their lubricant action, neither HA nor CS can effectively mitigate the osmotic imbalance induced by the disruption of the polyol cycle. Indeed, the elevated levels of glucose associated with hyperglycaemia lead to an osmotic insult, characterised by the accumulation of sorbitol and fructose while depleting the essential osmoprotectant INS [39]. This osmotic imbalance has detrimental effects, triggering apoptosis in ganglion cells and concurrently causing a reduction in nerve morphology and function (i.e., a reduction in their number and a reduction in their conduction velocities). These events significantly contribute to the development of diabetes-induced peripheral neuropathy [40,41,42,43]. INS has recently emerged as a potential therapeutic intervention in such conditions due to its osmoprotective action [44]. Therefore, replenishing INS levels seems to be useful in counteracting the osmotic insult inflicted by polyol cycle disruption.

It is worth mentioning that inositol depletion might also be directly involved in the etiopathogenesis of diabetes-induced dry eye. Indeed, it has been shown that it is possible to induce dry eye by blocking the inositol triphosphate (IP3) pathway by creating a knock-out animal model of the IP3 receptors (types 2 and 3). The KO mice displayed a reduced tear volume, reduced tear secretion, and a reduced lacrimal gland weight [45].

Notably, recent advancements in the management of diabetic keratopathy have underscored the significance of novel treatments aimed at restoring corneal nerve sensitivity [46,47,48]. Among these treatments, neurotrophic factors have emerged as promising candidates, as they play a crucial role in promoting cellular repair and mitigating the effects of diabetic neuropathy. For instance, studies have demonstrated that factors such as the brain-derived neurotrophic factor (BDNF) can enhance corneal epithelial health and sensitivity, addressing the underlying neuropathy that characterises the ocular complications in diabetic patients [6]. Furthermore, insulin-based therapies are being explored for their ability to facilitate corneal wound healing and metabolic regulation, potentially serving to reverse the impairments caused by hyperglycaemia [46,49].

It has to be mentioned that insulin is only one of the factors influencing corneal homeostasis; indeed, we should consider all insulin-like growth factor (IGF) systems, as there is evidence showing that insulin together with the IGF system is crucial for maintaining corneal health by regulating proliferation, differentiation, and wound healing across its cellular layers, including stromal repair. Disruptions in IGF signalling, such as in diabetes, impair wound healing and exacerbate complications. Emerging therapies, including topical insulin and IGF-1 analogues, show promise for restoring corneal health and function [19,46,50,51,52]. It is pivotal to mention that all the components of the IGF/insulin system signal via inositol second messengers; therefore, we can easily speculate that inositol depletion in diabetic subjects might be involved in the majority of the corneal alterations in diabetic patients by impairing the IGF/insulin system signal [53,54].

An increased sensitivity in diabetic patients upon the administration of CHA-CS could also be supported by CHA’s action, which has been shown to facilitate cellular and axonal ingrowth during the acellular fibrin matrix phase of peripheral nerve regeneration in a rat model [47] and support the improvement of corneal nerve fibre density and corneal sensation in a diabetic mouse model [48].

The results obtained by this study highlight the potential efficacy and safety of CHA-CS in the treatment of diabetes-induced corneal alteration. Due to its innovative formulation combining biotechnologically derived CS, CHA, and INS, it aims to not only alleviate patients’ ocular discomfort by its lubricant action, but also mitigate the effect of hyperglycaemia-induced neuropathy through the restoration of the osmotic balance in nerves. Moreover, preliminary in vitro results on wound healing underscore the potential of CHA-CS in corneal wound recovery.

It is worth noting that, on the basis of the present results and literature data, the same formulation tested in this study might be useful as an adjuvant therapy in combination with topical insulin in subjects affected by neurotrophic ulcers.

Despite the interesting results described in the present manuscript, this study has some limitations, such as the limited number of subjects included, that represent a further opportunity to improve the knowledge on the topic. To corroborate the present findings, a confocal microscopy study to analyse the corneal neuronal plexus would be beneficial, together with a corneal re-epithelization study on a diabetic animal model.

## 5. Conclusions

In conclusion, the present study provides valuable insights into the potential effectiveness of a novel ophthalmic solution containing CHA, CS, and INS for treating corneal sensitivity, and, therefore, function, in diabetic patient care. As the restoration of sensory function emerges as a critical element in preoperative care, it is imperative to develop targeted interventions that rehabilitate nerve health and optimise the conditions for successful graft acceptance and long-term ocular health.

## Figures and Tables

**Table 1:** Influence of the CHA-CS treatment on the wound closure (% of covered area) in the A549 and MRC5 cell lines. The mean values of wound closure (% of covered area) were derived from repeated experiments ± SD. * *p* ≤ 0.05, ** *p* ≤ 0.01, *** *p* ≤ 0.001, and **** *p* ≤ 0.0001 when comparing the CHA-CS treatment to the control group (Ctrl) (Student’s *t*-test).

	A549			MRC5		
Time (h)	Ctrl	CHA-CS	*p*-Value	Ctrl	CHA-CS	*p*-Value
2	1.981	4.367	0.158297	3.528	4.334	0.695281
4	9.626	10.33	0.647900	5.126	14.11 ****	0.000092
6	13.19	19.03 ***	0.000937	16.67	22.13 *	0.011207
22	54.94	61.01 ***	0.000614	82.05	91.15 ****	0.000065
24	60.91	70.41 ****	<0.000001	89.51	99.42 ***	0.000283
26	70.13	74.86 **	0.006420	---	---	---
28	73.61	84.08 ****	<0.000001	---	---	---
30	86.45	92.30 ***	0.000928	---	---	---

## Data Availability

The datasets presented in this article are not readily available due to privacy reasons. The data supporting the conclusions of this article will be made available by the authors upon request.

## Supplementary Materials

The following supporting information can be downloaded at: https://www.mdpi.com/article/10.3390/jcm14010245/s1. Figure S1: Representative picture of the wound-healing test.
