# Supplementary figures and images for: Enhancing Corneal Sensitivity in Diabetic Patients Through an Innovative Ophthalmic Solution: In Vivo and Vitro Results
Source: J Clin Med. 2025 Jan 3;14(1):245. doi: 10.3390/jcm14010245 (PMC11721187; doi:10.3390/jcm14010245)

Figure S1: Representative picture of the wound-healing test.

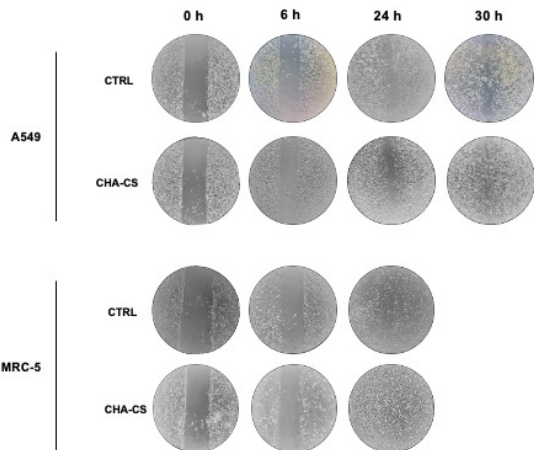

Supplement: Supplementary file 1 [file jcm-14-00245-s001.zip › jcm-3339729-supplementary.pdf]
